# l-Carnitine reduces reactive oxygen species/endoplasmic reticulum stress and maintains mitochondrial function during autophagy-mediated cell apoptosis in perfluorooctanesulfonate-treated renal tubular cells

**DOI:** 10.1038/s41598-022-08771-3

**Published:** 2022-03-18

**Authors:** Yuan-Chii Gladys Lee, Hsiu-Chu Chou, Yen-Ting Chen, Szu-Yu Tung, Tsui-Ling Ko, Batsaikhan Buyandelger, Li-Li Wen, Shu-Hui Juan

**Affiliations:** 1 Graduate Institute of Biomedical Informatics, College of Medical Science and Technology, Taipei Medical University, Taipei, Taiwan; 2 Department of Anatomy and Cell Biology, School of Medicine, College of Medicine, Taipei Medical University, Taipei, Taiwan; 3 Department of Physiology, School of Medicine, College of Medicine, Taipei Medical University, 250 Wu-Hsing Street, Taipei, 110 Taiwan; 4 College of Science, National Sun Yat-Sen University, Kaohsiung, Taiwan; 5 Department of Clinical Laboratory, En Chu Kong Hospital, New Taipei City, 237 Taiwan

**Keywords:** Biochemistry, Environmental sciences, Nephrology

## Abstract

We previously reported that perfluorooctanesulfonate (PFOS) causes autophagy-induced apoptosis in renal tubular cells (RTCs) through a mechanism dependent on reactive oxygen species (ROS)/extracellular signal-regulated kinase. This study extended our findings and determined the therapeutic potency of l-Carnitine in PFOS-treated RTCs. l-Carnitine (10 mM) reversed the effects of PFOS (100 µM) on autophagy induction and impaired autophagy flux. Furthermore, it downregulated the protein level of p47Phox, which is partly related to PFOS-induced increased cytosolic ROS in RTCs. Moreover, l-Carnitine reduced ROS production in mitochondria and restored PFOS-impeded mitochondrial function, leading to sustained normal adenosine triphosphate synthesis and oxygen consumption and reduced proton leakage in a Seahorse XF stress test. The increased inositol-requiring enzyme 1α expression by PFOS, which indicated endoplasmic reticulum (ER) stress activation, was associated with PFOS-mediated autophagy activation that could be attenuated through 4-phenylbutyrate (5 mM, an ER stress inhibitor) and l-Carnitine pretreatment. Therefore, by reducing the level of IRE1α, l-Carnitine reduced the levels of Beclin and LC3BII, consequently reducing the level of apoptotic biomarkers including Bax and cleaving PARP and caspase 3. Collectively, these results indicate that through the elimination of oxidative stress, extracellular signal–regulated kinase activation, and ER stress, l-Carnitine reduced cell autophagy/apoptosis and concomitantly increased cell viability in RTCs. This study clarified the potential mechanism of PFOS-mediated RTC apoptosis and provided a new strategy for using l-Carnitine to prevent and treat PFOS-induced RTC apoptosis.

## Introduction

Perfluorooctanesulfonate (PFOS) is ubiquitously distributed in our surrounding. It is extensively used in everyday items (e.g., Gore-Tex coats, fire extinguishers, and stainless pans) because of its hydrophobicity/hydrophilicity and stability. However, PFOS has attracted considerable attention because of its toxicity in various biological systems and to organisms including humans^1^. We previously demonstrated that PFOS can cause apoptosis through oxidative stress and the inflammation-related pathways in renal tubular cells (RTCs)^2^. In addition, PFOS can induce autophagy activation with increased Beclin and LC3BII expression through reactive oxygen species (ROS)-mediated extracellular signal–regulated kinase (ERK) activation, which is followed by impeded autophagic flux, increased lysosomal membrane permeability (LMP), and, consequently, RTC apoptosis. N-acetyl cysteine (a ROS scavenger) and U0126 (a mitogen-activated protein kinase [MAPK] inhibitor) can rescue PFOS-mediated autophagy activation and, consequently, autophagy-associated apoptosis in RTCs^3^.

Autophagy is a highly controlled self-protective catabolic mechanism that involves the degradation of unnecessary or dysfunctional components^4^. This process is highly controlled by autophagy-related (ATG) genes; it enables cells to cope with various forms of stress (e.g., nutrient deprivation, endoplasmic reticulum (ER) stress, pathogen infection, and hypoxia) and is considered a survival mechanism^5^. However, autophagy during prolonged stress can lead to cell death. ROS is a key regulator of both apoptosis and autophagy. In addition to ROS overproduction, the activation of MAPK and inactivation of phosphatidylinositol 3-kinase/Akt (a serine/threonine kinase that is also known as protein kinase B)/mammalian target of rapamycin (mTOR) are associated with both apoptosis and autophagy^6^. Similarly, numerous pathways are commonly used by these two processes in response to a single form of stress. Therefore, studies have explored the role of autophagy/apoptosis as a new target mechanism in domains such as chemo-resistant cancer therapy and toxicology.

L-trimethyl-3-hydroxy-ammoniabutanoate (l-Carnitine), a quaternary ammonium compound, is synthesized from methionine and lysine in cells^7^. Carnitines can remove excessive acyl groups from mitochondria as acylcarnitines through the carnitine acyltransferase pathway, which is crucial to maintaining normal mitochondrial function^8^. Mitochondrial function disturbance is a key event in numerous pathological conditions, such as hypoxic-ischemic injuries, stroke, and diabetes. Currently, l-Carnitine is clinically used as a supplement for patients on dialysis because these patients are susceptible to experiencing l-Carnitine reduction in plasma^9^. In addition, l-Carnitine increases the expression of antioxidative enzymes (e.g., eNOS, HO-1, and SOD), which in turn protects cardiomyocytes and endothelial cells from oxidative stress by reducing lipid peroxidation in cell membranes^10^. Furthermore, we previously revealed that l-Carnitine can alleviate gentamicin-induced apoptosis in RTCs through PPARα activation in a prostaglandin I_2_-dependent manner^11^. PPARγ activation by l-Carnitine reduces hypertension-associated renal fibrosis^12^. Relatedly, our group demonstrated that the antioxidative and anti-inflammatory properties of l-Carnitine protect RTCs from PFOS-mediated renal injury^2^ and fibrosis through a PPARγ-dependent mechanism^13^.

Endoplasmic reticulum (ER) stress is a secondary response caused by oxidative stress or inflammation mediated by aggregated proteins^14,15^. ER stress activation can mitigate protein synthesis, induce ER chaperones to facilitate protein folding, and increase ER-associated degradation to reduce protein load. Furthermore, a mechanistic link between ER stress and autophagy suggests that ER stress and unfolded protein response pathways can induce the expression of autophagy-related protein by activating ER stress sensors^16^. Therefore, autophagy is induced to assist the degradation of damaged proteins by lysosome enzymes through autolysosome formation. However, this process is followed by apoptosis if the activation of ER stress and autophagy fails to prevent cell damage.

In the present study, we further explored the involvement of ER stress in autophagy-mediated apoptosis in PFOS-treated RTCs; in addition to the antioxidative activity of l-Carnitine, its protective effect may be associated with ER stress inhibition, ERK inactivation, and the maintenance of normal mitochondrial function.

## Materials and methods

### Cell culture and reagents

Rat renal proximal tubular cells (RTCs, NRK-52E) were purchased from the Bioresource Collection and Research Center (Hsinchu, Taiwan), and culture cells in Dulbecco’s modified Eagle’s medium (DMEM) were supplemented with an antibiotic/antifungal solution and 5% fetal bovine serum (FBS, pH 7.2). Cells were grown to 85%–95% confluence before they were placed in a humidified 37 °C incubator, and cells from passages 5–20 were used. FBS, DMEM, and tissue culture reagents were obtained from Invitrogen (Carlsbad, CA, USA). PFOS and 4-phenylbutyrate (4-PBA) were purchased from Sigma Chemical (St. Louis, MO, USA). Oligomycin, carbonyl cyanide m-chlorophenyl hydrazone (CCCP), and rotenone were obtained from Merck Millipore (Darmstadt, Germany); 3-(4,5-dimethyl thiazol-2-yl)-2,5-diphenyl tetrazolium bromide (MTT) was obtained from SERVA Electrophoresis (Berlin, Germany). l-Carnitine and U0126 were obtained from Healthmate (Taichung, Taiwan) and Tocris Cookson (Bristol, UK), respectively. Protein assay agents were purchased from Bio-Rad (Hercules, CA, USA). The chemical concentration and treatment duration for each assay were set in accordance with the protocols used in our previously published studies^17–19^ or pilot studies.

### Analysis of apoptosis, cell cycle, and ROS detection in flow cytometry

The aforementioned cells were pretreated with l-Carnitine for 24 h and then subjected to PFOS challenge at specific time points. The resulting cells were harvested through centrifugation at 800 rpm for 5 min and then washed twice with ice-cold phosphate-buffered saline (PBS). Apoptosis induced by PFOS in RTC cells was detected through Annexin V-FITC/PI double labeling flow analysis (Elabscience, Houston, Texas USA), which was conducted in accordance with the manufacturer’s instructions. In our cell-cycle analysis, the cells were seeded in 60-mm culture dishes and then serum-deprived for 24 h, after which the indicated treatment was administered. They were fixed through slow vortexing with 3 mL of alcohol (70%) and then stored at − 20 °C overnight. One milliliter of a PBS-based reaction mixture containing propidium iodide (20 μg/mL), RNase (0.2 μg/mL), and 0.1% Triton X-100 (Sigma) was added to the cell samples at a density of 1 × 10^6^ cells/mL and incubated in the dark for 40 min before a DNA content analysis was conducted through flow cytometry. To detect cytosolic and mitochondrial ROS, the cells (5 × 10^5^) that were seeded in 60-mm dishes with indicated treatments were prepared and stained with MitoSox (5 μM; Thermo Fisher Scientific, Waltham, MA USA) and 2′,7′-dichlorofluorescein diacetate (DCFDA, 2.5 μM, Sigma) for 15 min in 0.5 mL of PBS. The excitation/emission wavelength was 488 nm/610 ~ 620 nm for MitoSox and 488 nm/525 ~ 540 nm for DCFDA. Flow cytometry was performed using CytoFLEX and CytoExpert software (Beckman Coulter); dot plots and histograms were used to visualize data.

### MTT assay

Cell viability was assessed by examining the activity of mitochondrial dehydrogenase, which reduces MTT to formazan. In brief, cells were seeded in 24‐well plates at a density of 1.5 × 10^4^ cells/well and treated with dimethyl sulfoxide (DMSO) or 100 µM PFOS for 24 h with or without pretreatment with 10 mM l-Carnitine for 24 h. MTT was then added to each well, and incubation was conducted for 30 min at 37 °C. The formazan that was formed was dissolved in DMSO for 30 min on a shaker, and absorbance was measured using a spectrophotometer at a wavelength of 570 nm.

### Chemical fluorescence dyes for detecting cytosolic and mitochondrial ROS and lysosomal membrane stability in PFOS-treated RTCs

The cells that were grown overnight on glass coverslips in six-well Petri dishes were pretreated with 10 mM l-Carnitine for 24 h with or without an additional treatment with 100 µM PFOS for 24 h. For cytosolic ROS detection, the resulting cells were stained with 2.5 µM DCFDA. For mitochondrial ROS detection, the cells were stained with 5 µg/mL MitoSox dye (Thermo Scientific, Waltham, MA, USA) at 37 °C for 15 min, followed by three PBS washes. The DCFDA was oxidized by ROS to form dichlorofluorescein, which is a fluorescent compound that can be detected using a fluorescence microscope at an excitation/emission of 495/529 nm^20^. The mitochondrial superoxide that was detected using MitoSox reagent was observed through a fluorescence microscope at an excitation/emission of 510/580 nm (red color). The resulting cells were fixed in 4% paraformaldehyde and incubated with a blocking buffer for 1 h, and the cells were permeabilized with 0.2% Triton and 0.1% Tween 20 in a blocking buffer (3% bovine serum albumin in PBS) for 2 h. Furthermore, the MitoSox-stained cells were counterstained to identify nuclei by using the 4′,6-diamidino-2-phenylindole (DAPI) dye. Additionally, during the acridine orange (AO) reaction for a lysosome membrane permeability assay, the cells with indicated treatments were stained with 2 µg/mL AO (Invitrogen) for 15 min at 37 °C; the presence of red stains indicated that the AO reagent reacted with intact lysosome with an acidic pH value of approximately 4.5–5.0, whereas the presence of green stains indicated increased LMP or an increased lysosome pH value in the reaction. Fluorescence was viewed using a charge-coupled device camera (DP72, Olympus, Melville, NY, USA) attached to a microscope system (BX51, Olympus) at 100 × magnification. Four coverslips were examined in each experimental group. The subsequent procedures have been described in a previous study^21^.

### Transfection of fluorescent LC3 constructs

The plasmids of pEX-GFP-hLC3WT and Ds-RFP-GFP-LC3 (pQCXI Puro DsRed-LC3-GFP) used in the present study were provided by Drs. Chun-Han Chen and Ching-Hao Li (Taipei Medical University, Taipei, Taiwan), who purchased them from Addgene (Cambridge, MA, USA). Lipofectamine 2000 (Invitrogen, Carlsbad, USA) was used to deliver plasmid constructs (2.2 μg/6.0-cm Petri dish) into cells in accordance with the manufacturer’s instructions. Cells transfected with pEX-GFP-hLC3WT were selected with G418 for use as permanent clones of GFP-hLC3WT overexpression. After transfection, the cells were plated in DMEM with 5% FBS and 400 mg/mL G418 for a month. Additionally, cells transfected with the plasmid of Ds-RFP-GFP-LC3 (Ds-LC3) overnight were pretreated with l-Carnitine for 24 h and subsequently subjected to an additional 24-h PFOS treatment. The increased GFP/RFP-positive puncta represent increased autophagosome formation. However, the RFP-positive/GFP-negative puncta represent the fusion of autophagosome to lysosome to form autolysosome, during which GFP is quenched because of its susceptibility to a low pH in lysosome. Fluorescence was viewed using a Zeiss Axio Observer Z1 inverted phase contrast microscope (Wilmington, MA, USA).

### Seahorse MitoStress assay

RTCs with a density of 6000–12,000 cells/well were seeded in XF24-well microplates overnight and subsequently subjected to a 24-h treatment with increasing concentrations of PFOS (100–200 µM) or an additional 24 h of l-Carnitine pretreatment. A fluorescent probe was activated by immersing the microplates in a calibration solution for 1 day; these plates were placed in a CO_2_-free incubator at 37 °C overnight before an assay was performed. The fluorescent/detective probe was activated using the reagent supplied with the probe and in accordance with the manufacturer’s instructions. The cell growth medium was replaced by a medium containing 2% FBS DMEM without sodium bicarbonate 1 h before the Seahorse MitoStress assay was performed. Compounds of oligomycin (10 µM), carbonyl cyanide m-chlorophenyl hydrazone (40 µM), and rotenone (5 µM) were prepared and loaded into XFe24 sensor cartridges from port A to C for sequential injection over time. Oxygen consumption rate (OCR), spare respiratory capacity, and proton leakage were automatically calculated and recorded using the Seahorse XFe24 analyzer (Santa Clara, CA, USA)^22^.

### Western blot analysis

RTCs were seeded in 6- or 10-cm dishes after completion of the indicated treatments. The resulting cells were harvested for total cell lysates with the addition of protease inhibitors in accordance with the manufacturer’s instructions. The cell lysates were centrifuged at 16,000×*g* and 4 °C for 20 min; the supernatants containing total proteins were collected, and the protein concentration was quantified using a protein assay dye (Bio-Rad). Antibodies for BECN1 (Beclin), Bax, pERK1/2 (T202/Y204, T185/Y187), PARP/cleaved PARP, p47Phox, IRE1α, pAKT (S473) and β-actin (1:500, Santa Cruz Biotechnology, Santa Cruz, California, USA); microtubule-associated protein light chain (LC) 3B and nucleoporin p62/SQSTM1 (1:500, GeneTex, Irvine, California, USA); and caspase 3, pmTOR (S2448) and VDAC (1:1000, Cell Signaling Technology, Danvers, MA, USA) were included in the assay. The cell lysates (80 µg) were electrophoresed on a 10%–12% sodium dodecyl sulfate–polyacrylamide gel and then transblotted onto a Hybond-P membrane (GE Healthcare, Hong Kong). The membranes were blocked with 5% skimmed milk for 1 h at room temperature and then incubated with the desired primary antibodies and appropriate horseradish peroxidase–conjugated (HRP) secondary antibodies. Subsequently, an immobilon western chemiluminescent HRP substrate (Merck Millipore, Darmstadt, Germany) was used for detection in accordance with the manufacturer’s instructions. The expected protein bands were detected using the UVP BioSpectrum 500 imaging system (Analytik Jena US, Upland, CA, USA). The relative abundance of the target protein was measured using the ImageJ software and normalized to the intensity of the β-actin band. The subsequent procedures are described in a previous study^23^.

### Statistical analysis

Data from at least three experiments are presented as means ± standard errors of mean (SEM). *P* values for differences in the means between two conditions were calculated using unpaired Student’s *t* tests, and the differences between groups were tested using one-way analysis of variance or Bonferroni post hoc test. A *P* value of < 0.05 was considered significant.

## Results

### l-Carnitine attenuated the increased ROS levels in the cytosol and mitochondria of PFOS-treated RTCs and the cell death of such cells

**Figure 1:**
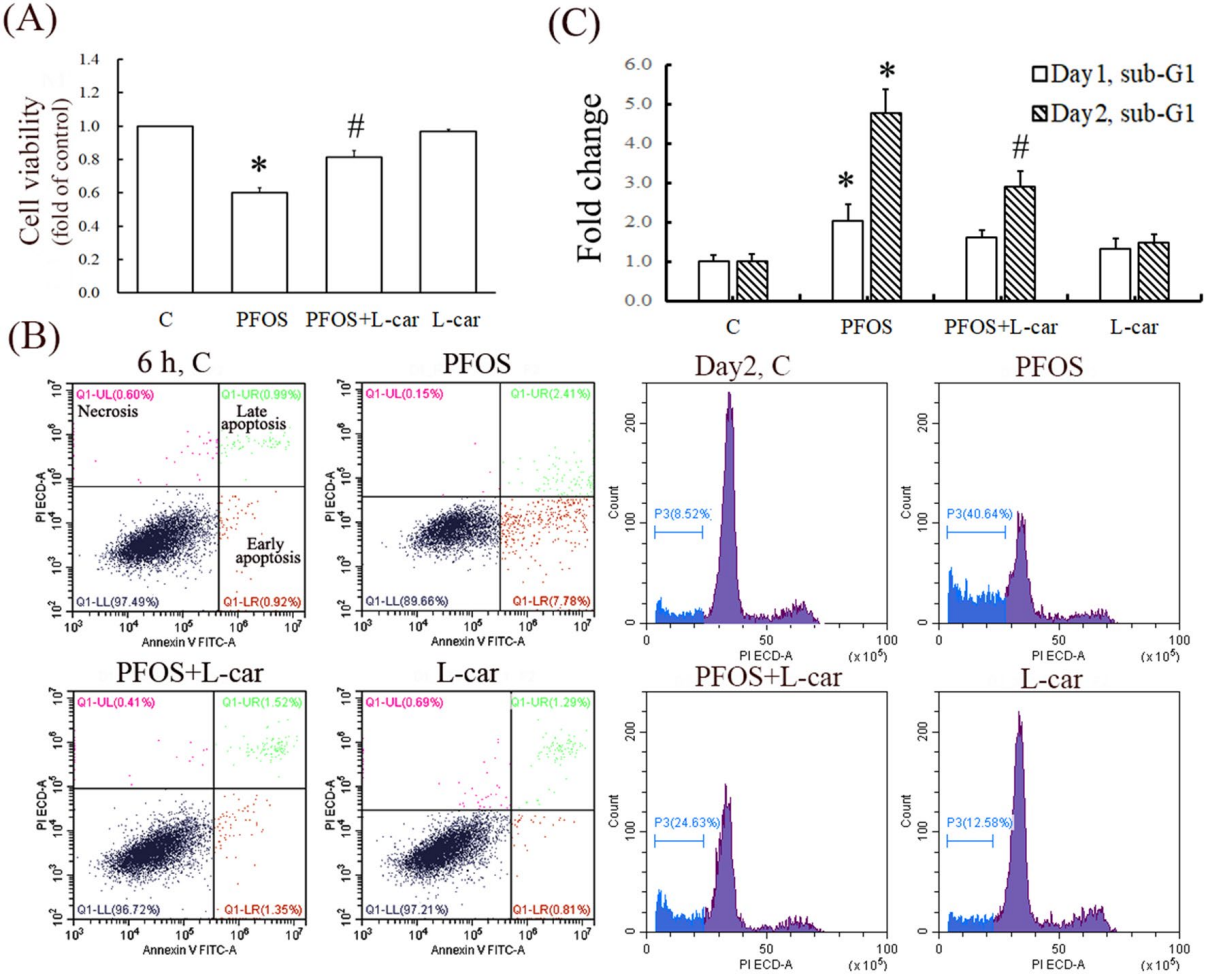
Attenuation of perfluorooctanesulfonate (PFOS)-mediated decrease in cell viability through l-Carnitine in renal tubular cells (RTCs). (**A**) Effect of l-Carnitine on reduction of RTC viability through PFOS insult was analyzed through 3-(4,5-dimethyl thiazol-2-yl)-2,5-diphenyl tetrazolium bromide (MTT) assay. In total, 1.5 × 10^4^ cells/well were seeded in a 24-well plate; this was followed by 24 h of PFOS challenge with or without 24 h of l-Carnitine pretreatment. (**B**) Cells pretreated with l-Carnitine overnight were then subjected to 6-h PFOS treatment. Harvested cells were double-stained with Annexin V-PI for flow cytometric analysis of apoptosis. The results are representative of three independent experiments. (**C**) Cells were incubated in 0.5% serum Dulbecco’s modified Eagle’s medium (DMEM) for 24 h to induce quiescence; this was followed by 10-µM PFOS treatment for 24 and 48 h with or without 24 h of 10-mM l-Carnitine pretreatment in 5% fetal bovine serum (FBS) DMEM medium. The percentages of the sub-G1 phase and the cell cycle were analyzed through flow cytometry analysis. The cell number is presented on the *y*-axis, and PI energy-coupled dye labeling (DNA content) information is presented on the *x*-axis. The results of a representative experiment are presented. The data are representative of the results of three independent experiments, and they are presented as means ± SEM (*P < 0.05 vs. control; ^#^*P* < 0.05 vs. PFOS alone).

**Figure 2:**
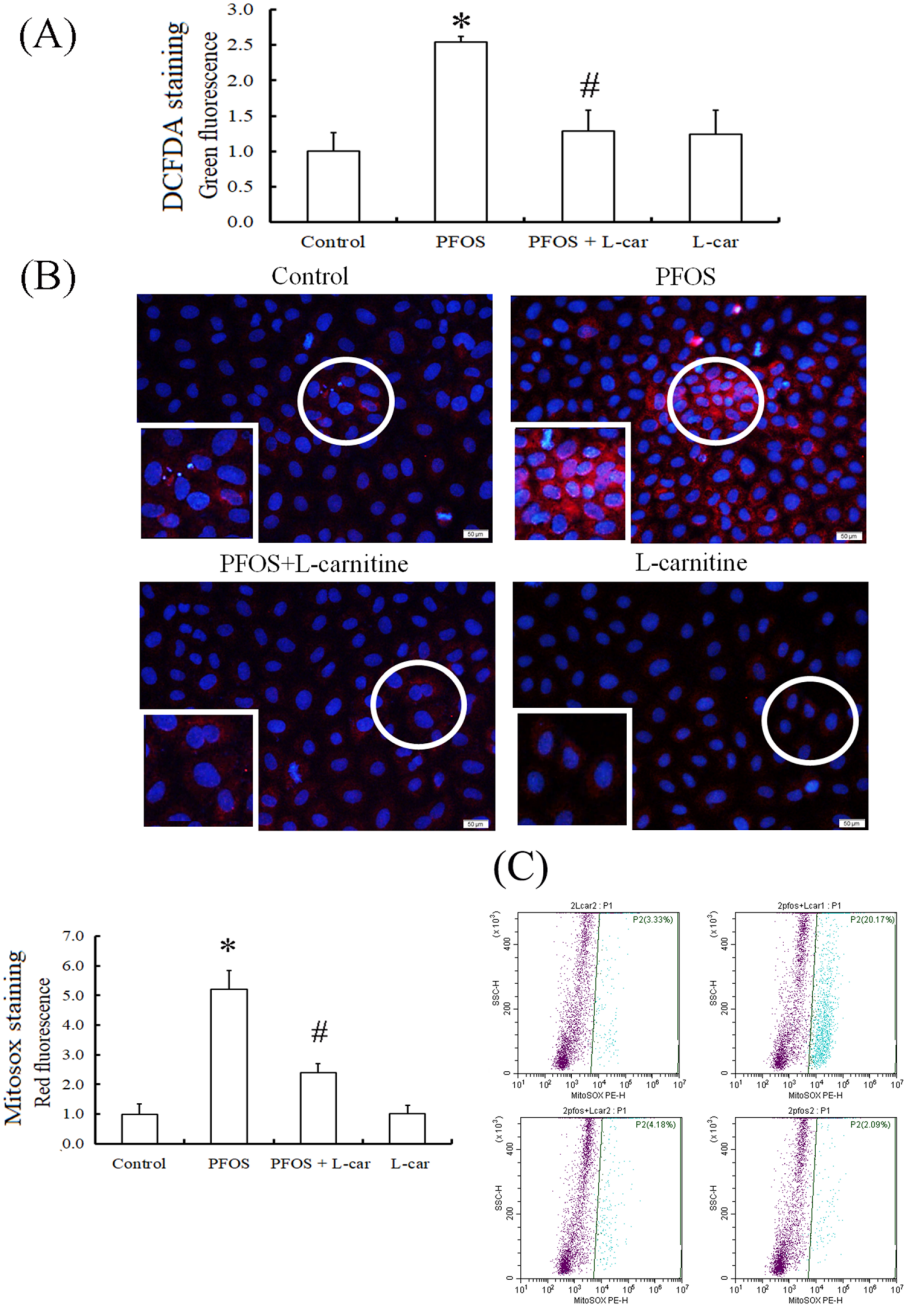
Reduction of cytosolic and mitochondrial reactive oxygen species (ROS) through l-Carnitine in perfluorooctanesulfonate (PFOS)-treated renal tubular cells (RTCs). ROS induction through PFOS was detected in cytosol and mitochondria after 24-h treatment. (**A**) The effect of 24-h PFOS treatment on cytosolic ROS production was assessed through 2.5 µM 2′,7′-dichlorofluorescin diacetate (DCFDA) staining and intervened by performing 24 h of 10-mM l-Carnitine pretreatment. (**B**) Mitochondrial superoxide was measured using MitoSox Red (Molecular Probes) at an excitation of 510 nm and emission of 580 nm. The effect of PFOS on mitochondrial ROS production was investigated using MitoSox staining, followed by 5 min of DAPI staining for nuclei counterstain; effects were evaluated through l-Carnitine pretreatment of PFOS-treated RTCs for 24 h. The image displays representative images obtained using a fluorescence microscope (100 × magnification; scale bar, 50 μm). The cells in the white squares on the lower left sections of the panels are magnified images of those in the white circles. The data are representative of the results of three independent experiments. The quantified data are plotted in bar graphs and presented as means ± SEM (*P < 0.05 vs. control; ^#^*P* < 0.05 vs. PFOS treatment alone). (**C**) To evaluate the production of mitochondrial ROS, RTCs pretreated with l-Carnitine for 24 h were followed by a 5-h PFOS treatment. The resulting cells were stained with 5-µM MitoSox for 15 min and then detected through flow cytometry with Ex/Em wavelengths of 488/610 nm. The results are representative of four independent experiments.

We previously demonstrated that N-acetylcysteine (NAC), a ROS scavenger, rescues RTCs from PFOS-mediated apoptosis triggered by autophagy activation because the autophagy inhibition due to 3-methyladenine can block the aforementioned apoptosis^3^. Herein, we examined the mechanism underlying the protective effects of l-Carnitine on autophagy-associated apoptosis in the RTCs treated with PFOS. The PFOS-mediated decrease in cell viability could be significantly reversed through additional l-Carnitine treatment, as evidenced by the increased cell viability observed using the MTT assay (Fig. 1A). In the flow analysis with Annexin-V-PI double staining, PFOS increased early (lower right) and late (upper right) cell apoptosis by approximately 10%, which was substantially reduced to 2.8% through the administration of additional l-Carnitine pretreatment to RTCs at the 6-h point of their PFOS treatment (Fig. 1B). Additionally, PFOS increased the subG1 phase of the cell cycle by up to 2–5-fold relative to the control on Days 1 and 2 of the treatment (upper panel of Fig. 1C), whereas l-Carnitine significantly reduced it by 60% on Day 2 (Fig. 1C). Furthermore, the antioxidative activity of l-Carnitine in PFOS-induced RTCs was analyzed (Fig. 2); a decrease in PFOS-induced ROS by l-Carnitine was observed in the cytosol and mitochondria of the treated cells through the DCFDA reaction (Fig. 2A) and MitoSox detection (Fig. 2B), respectively. Additionally, a MitoSox-based cytometric analysis was employed to detect mitochondrial ROS formation in RTCs. The results presented in Fig. 2C reveal that PFOS increased mitochondrial ROS formation by up to 20% in the MitoSox-positive cells, and this figure could be substantially attenuated to less than 5% through l-Carnitine treatment.

### l-Carnitine pretreatment reversed impaired mitochondrial bioenergetics function caused by PFOS in RTCs

**Figure 3:**
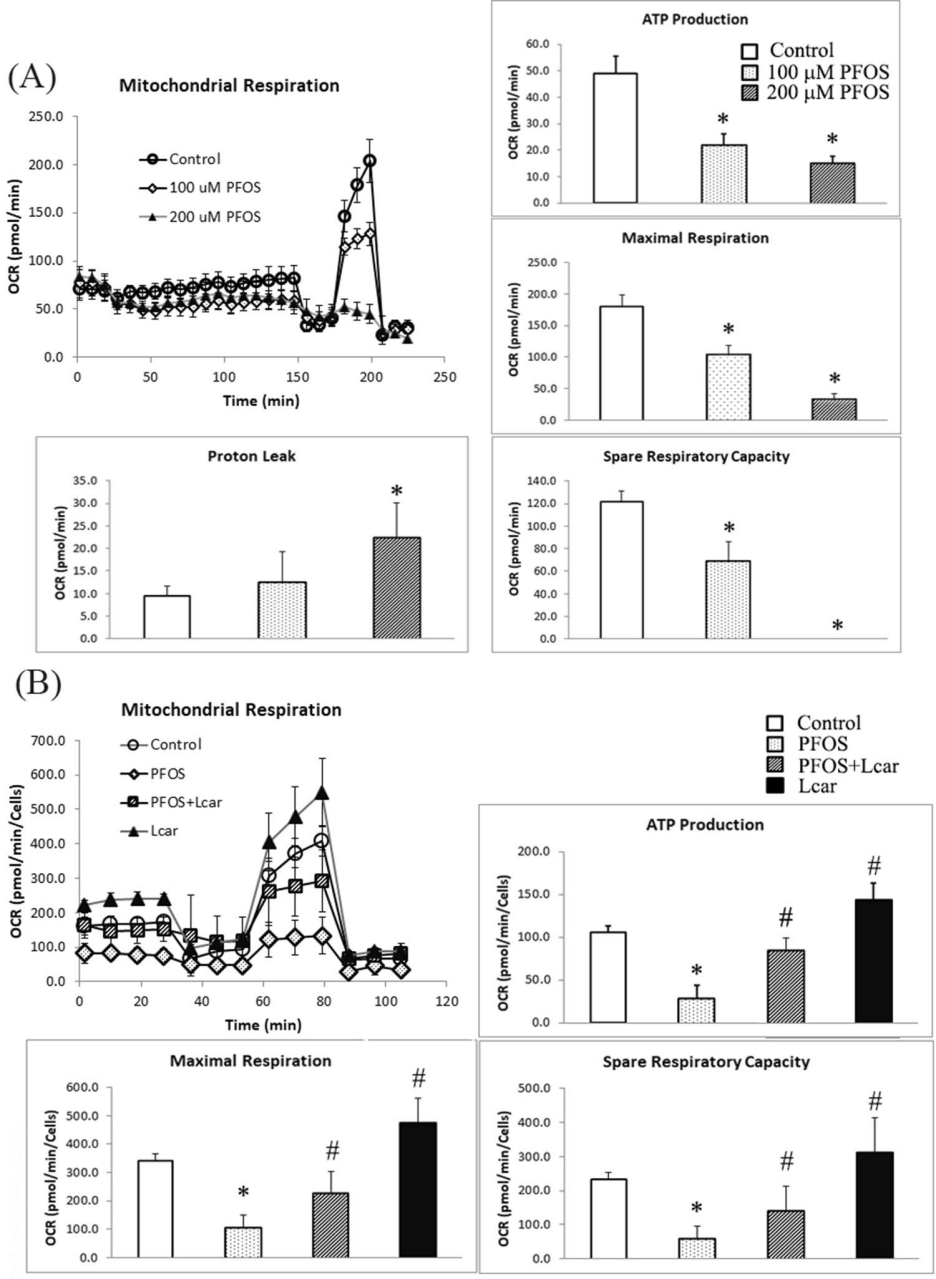
Protective effect of l-Carnitine on mitochondrial function impaired by perfluorooctanesulfonate (PFOS) in a Seahorse MitoStress assay. (**A**) Cells were cultured overnight in XFe24-well microplates with 12,000 cells/well; this was followed by increased concentrations of 100- and 200-µM PFOS challenges for 24 h. (**B**) To examine the intervention by l-Carnitine, the seeded 6000 cells/well were pretreated with l-Carnitine for 24 h and then subjected to a 100-µM PFOS challenge for another 24 h. The ports on sensor cartridges were step-wise loaded with tenfold concentrated compounds from ports A to C (specifically, 10 µM oligomycin for port A, 40 µM carbonyl cyanide m-chlorophenyl hydrazone for port B, and 5 µM rotenone for port C). The data are presented as means ± SEM of six independent experiments. A value of *P < 0.05 was considered statistically significant (*P < 0.05 vs. normal control; ^#^*P* < 0.05 vs. the PFOS group).

We previously demonstrated that l-Carnitine reduces oxidative stress by increasing the expression of Gpx-1, SOD, and catalase in PFOS-treated RTCs^2^. Consistent with our finding, studies have reported that l-Carnitine maintained normal mitochondrial function in response to various PFOS challenges^24,25^. Therefore, the protective effect of l-Carnitine on mitochondria was further evaluated using the Seahorse XF Cell Mito Stress test. We first examined the concentration-dependent effect of PFOS on mitochondrial bioenergetics and discovered that an increase in the PFOS concentration from 100 to 200 µM significantly decreased mitochondrial bioenergetics, as evidenced by the decreased ATP production, maximal respiration and spare respiratory capacity, and increased proton leakage (Fig. 3A). By contrast, l-Carnitine pretreatment could reverse PFOS-induced mitochondrial function impairment by repairing mitochondrial biogenesis, which was achieved by increasing the ATP production, maximal respiration, and spare respiratory capacity in the RTCs treated with 100-µM PFOS (Fig. 3B).

### l-Carnitine protected RTCs from PFOS-mediated apoptosis by sequentially inhibiting the induction of ROS, pERK1/2, IRE1α, and autophagy

**Figure 4:**
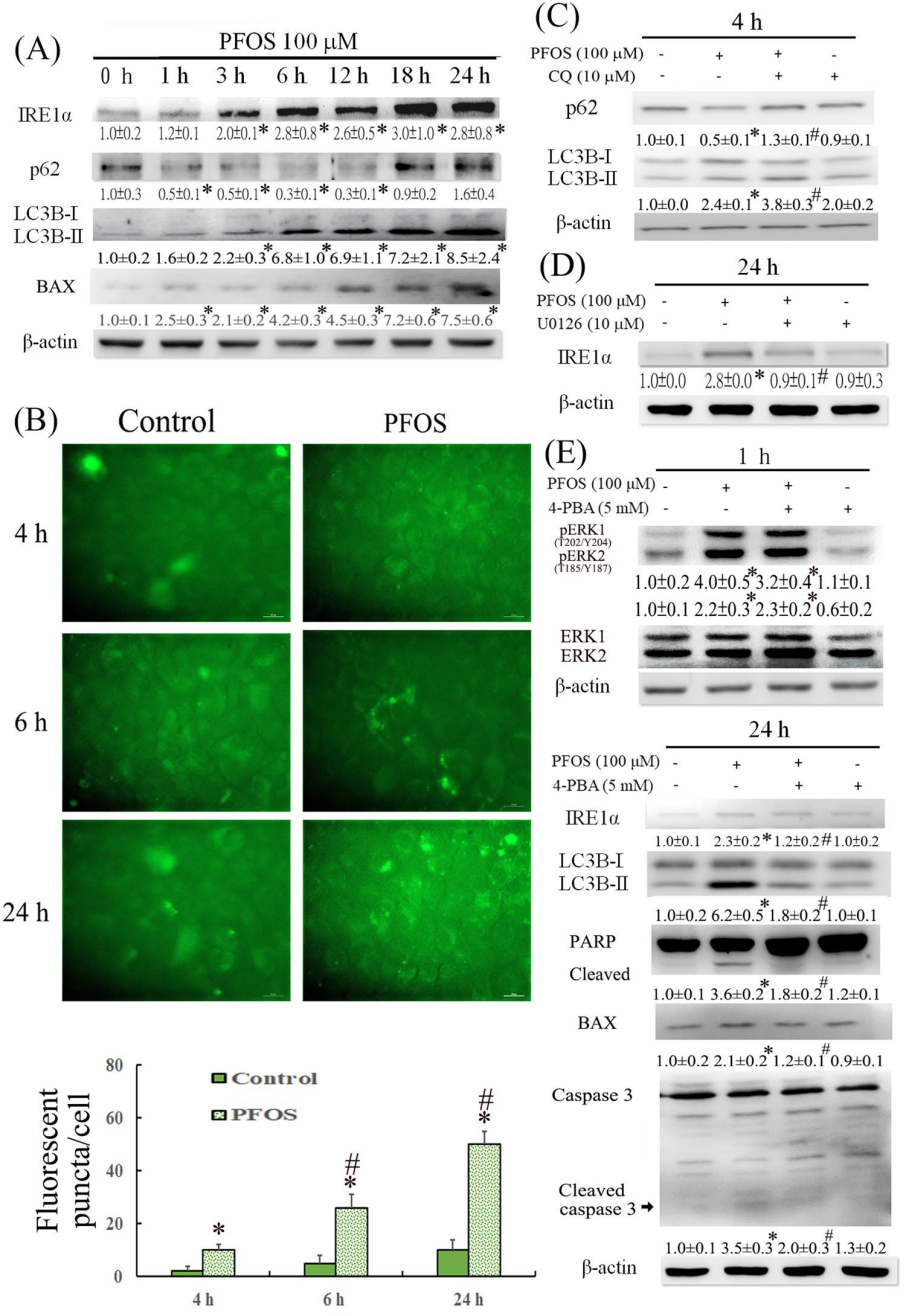
Time-dependent effect of perfluorooctanesulfonate (PFOS) on autophagy induction and impaired autophagy flux through an ERK and ER stress-dependent mechanism. (**A**) Cells treated with 100 µM PFOS for 1, 3, 6, 12 18, and 24 h were harvested and analyzed through a Western blot analysis for proteins involved in autophagy processes, including IRE1α (an ER stress marker), p62, and LC3BII (autophagy). (**B**) The clone of renal tubular cells (RTCs) with LC3B-GFP overexpression was subjected to 4–24 h of PFOS treatment. The images of LC3B-GFP positive cells were taken under a fluorescent microscope. The quantitated data are plotted using bar graphs and presented as means ± SEM (*P < 0.05 vs. 4 h control; ^#^*P* < 0.05 vs. 4 h PFOS) (**C**) Chloroquine was added to cells treated with PFOS for 4 h during the final hour of incubation, after which a Western blot analysis was performed. The causal relationship between the activation of the ERK pathway and ER stress on autophagy induction by PFOS was investigated using 10 µM U0126 (a MAPK kinases inhibitor) (**D**) and 5 mM 4-PBA (an ER stress inhibitor) (**E**) to examine their effects on the induction of IRE1α, pERK1/2, or LC3BII at 1- or 24-h PFOS treatment by using a Western blot analysis. The intensity of each protein band was quantitated through densitometry and normalized with the specific endogenous protein or an internal control of β-actin, and the values are presented as the means ± SEM of each membrane blot (*P < 0.05 vs. control; ^#^*P* < 0.05 vs. PFOS alone). The data are representative of the results of three independent experiments. The gels have been run in the same experimental conditions and the cropped blots were shown. The entire gel pictures were shown in the Supplemental Fig. 1.

The time-dependent effect of PFOS on the autophagy process was evaluated (Fig. 4A), and the results revealed an upregulation of IRE1α (an ER stress marker), indicating ER stress activation, reduced p62 levels, and increased LC3BII levels within 0–12 h of PFOS treatment. After 18–24 h of PFOS treatment, a significant induction of IRE1α and LC3BII was observed, whereas the p62 level was increased; these results suggested the impairment of autophagic flux in RTCs. To ensure autophagic activation by PFOS, we generated a stable GFP-LC3B clone of RTCs and counted LC3 puncta at 4–24 h of PFOS treatment (Fig. 4B); PFOS significantly increased the numbers of LC3 puncta in a time-dependent fashion. Furthermore, to evaluate the blockade of autophagic flux by PFOS, lysosomal inhibitor chloroquine (CQ) was added to PFOS-treated RTCs during the final hour of the 4-h PFOS treatment; the results of the Western blot analysis revealed that the PFOS-mediated p62 downregulation due to autophagy activation can be restored through additional CQ treatment, whereas the level of LC3BII can be further increased by the addition of CQ to RTCs (Fig. 4C).

**Figure 5:**
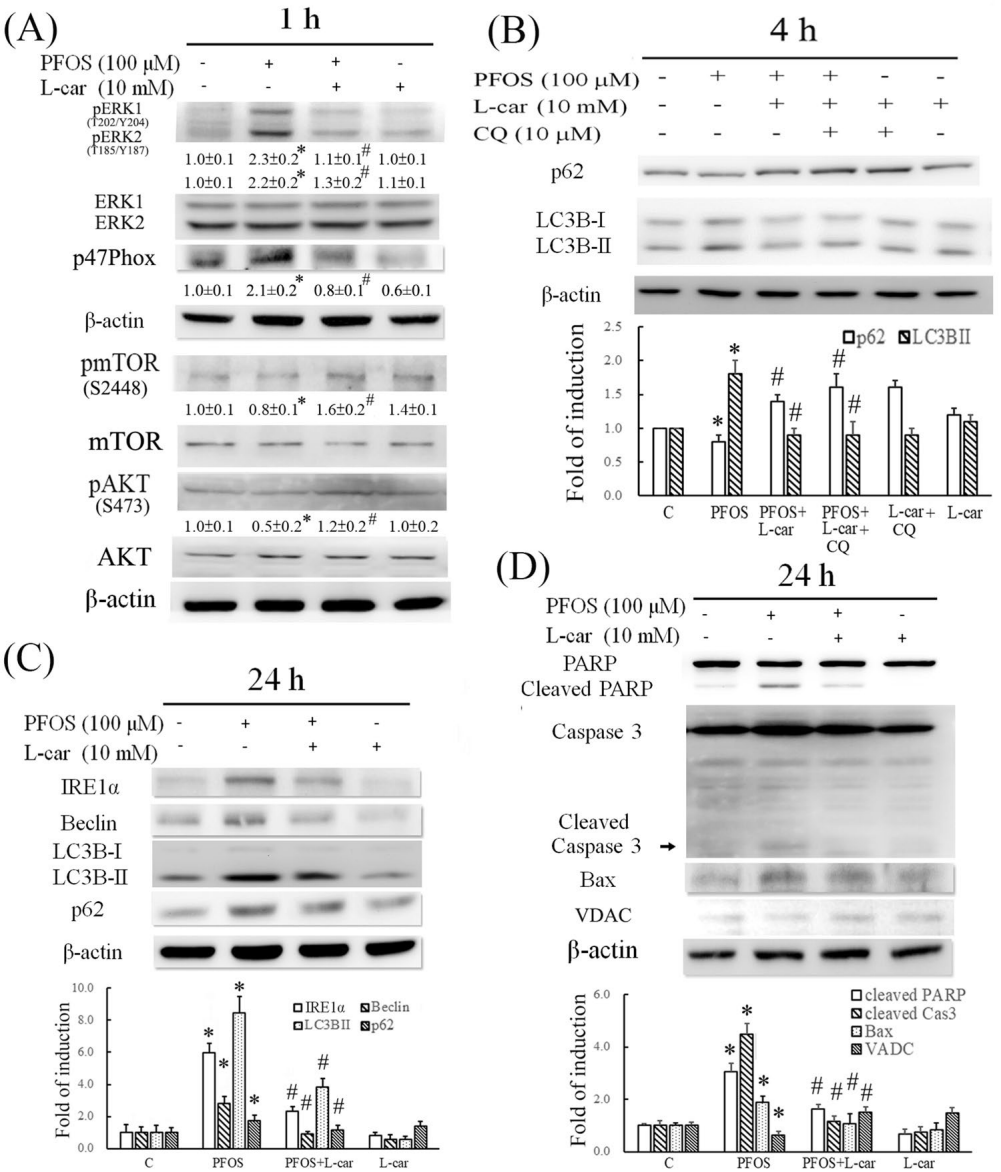
Protective effect of l-Carnitine on perfluorooctanesulfonate (PFOS)-induced autophagy-associated apoptosis through its antioxidative activity. (**A**) Renal tubular cells (RTCs) were pretreated with l-Carnitine for 24 h; this was followed by 1 h of PFOS treatment to examine the induction of pERK1/2, p47Phox, pmTOR, and pAKT levels through a Western blot analysis. The intensity of each protein band was quantified through densitometry and normalized with the specific endogenous protein or an internal control of β-actin, and the values are presented as the means ± SEM of each membrane blot (*P* < 0.05 vs. control; ^#^*P* < 0.05 vs. PFOS alone). (**B**) The effect of l-Carnitine on attenuating the blockade of autophagic flux through PFOS was evaluated in the presence of chloroquine during the final hour of the 4-h PFOS treatment. A Western blot analysis of p62 and LC3BI/II was performed. (**C**, **D**) Cells treated with PFOS for 24 h were examined for the biomarkers of ER stress (e.g., IRE1α), autophagy (e.g., Beclin and LC3BII), and apoptosis (e.g., Bax, cleaved PARP and caspase 3). The intensity of each protein band was quantitated through densitometry and normalized with an internal control of β-actin, and the values are presented as the means ± SEM of each membrane blot (*P < 0.05 vs. control; ^#^*P* < 0.05 vs. PFOS alone). The data are representative of the results of three independent experiments. The gels have been run in the same experimental conditions and the cropped blots were shown. The entire gel pictures were shown in the Supplemental Fig. 1.

The results presented in Fig. 4A indicate the concentration-dependent induction of IRE1α, suggesting the involvement of ER stress activation in the autophagy-associated apoptosis in PFOS-treated RTCs. Thus, the causal relationship among ER stress induction, pERK1/2, and autophagy activation was examined using MAPK/ERK and ER stress inhibitors, namely U0126 and 4-PBA. The blockade of ERK1/2 activation by U0126 (a MAPK kinases inhibitor) attenuated the IRE1α level, suggesting that ERK1/2 activation causes ER stress activation in PFOS-treated RTCs (Fig. 4D). Conversely, the ER stress inhibitor (4-PBA) did not mitigate PFOS-phosphorylated ERK1/2 levels at 1 h of PFOS treatment in RTCs, but it reduced the level of LC3BII (an autophagy biomarker) at 24 h of PFOS treatment in RTCs (Fig. 4E). Therefore, the PFOS-mediated apoptosis started with ERK1/2 activation, which was followed by ER stress and autophagy induction.

l-Carnitine reduced cytosolic ROS production in PFOS-treated RTCs (Fig. 2), and its underlying mechanism was analyzed for p47Phox, which was examined to evaluate the protective effect of l-Carnitine on the level of p47Phox induced by PFOS treatment through a Western blot analysis. l-Carnitine significantly reversed the increased p47Phox level, which could result in cytosolic ROS production and, subsequently, ERK1/2 phosphorylation at 1 h of PFOS treatment (Fig. 5A). In addition, the upstream signaling of autophagy induction (e.g., pmTOR and pAKT) was examined in PFOS-treated RTCs with and without additional l-Carnitine pretreatment. PFOS reduced the phosphorylated levels of mTOR and AKT and induced autophagy activation; however, the effects of autophagy activation could be reversed with additional l-Carnitine treatment, which led to autophagy inhibition in PFOS-treated RTCs (Fig. 5A). This provided additional signaling evidence regarding autophagy induction due to PFOS and its alleviation through additional l-Carnitine treatment. The effect of l-Carnitine on the attenuated autophagic flux induced by PFOS was further evaluated using an additional CQ treatment. l-Carnitine reversed the effect of 4 h of PFOS treatment, leading to increased p62 and reduced LC3BII levels. The additional CQ treatment produced similar outcomes but through other mechanisms; CQ reversed the effects of a short 4-h PFOS treatment in reducing p62 and increasing LC3BII levels by blocking lysosome activity (Fig. 5B).

Moreover, the addition treatment of l-Carnitine eliminated the upregulation of IRE1α (a biomarker of ER stress), Beclin, and LC3BII (a biomarker of autophagy) in the RTCs treated with PFOS for 24 h (Fig. 5C). Furthermore, a Western blot analysis revealed that l-Carnitine attenuated the increased p62 accumulation caused by 24-h PFOS treatment (Fig. 5C), indicating its protective effect in mitigating the impaired autophagic flux in the RTCs treated with PFOS. Consequently, the PFOS-mediated activation of apoptosis markers (i.e., Bax, cleaved PARP, and caspase 3) was attenuated, whereas the reduced mitochondrial marker (i.e., VDAC) was restored through additional l-Carnitine treatment (Fig. 5D); these results were also revealed through the Western blot analysis.

### Protective effect of l-Carnitine on maintaining lysosomal membrane stability (as determined using an AO assay and tandem GFP-RFP-LC3 autophagy flux reporter)

**Figure 6:**
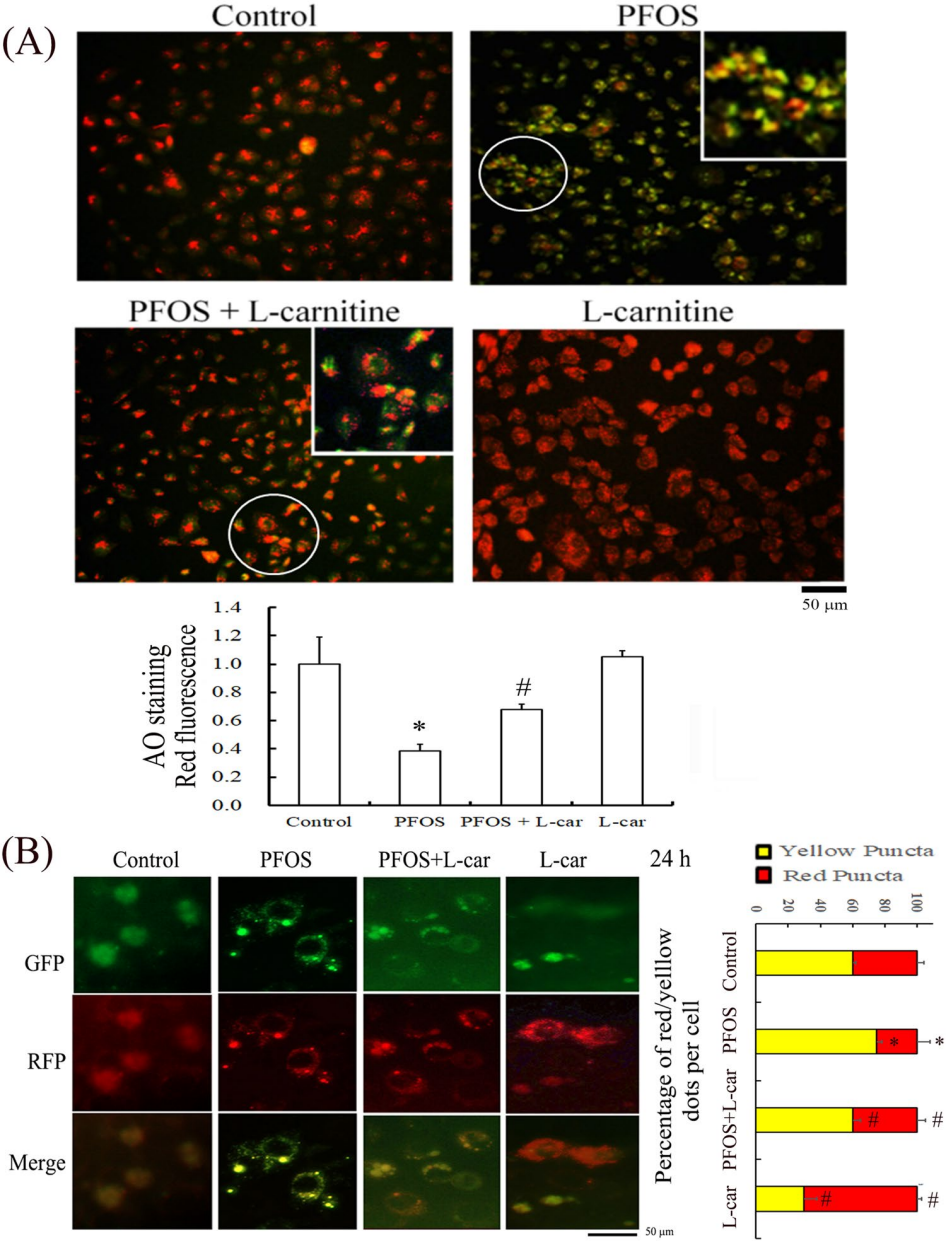
Perfluorooctanesulfonate (PFOS)-mediated increase in lysosome membrane permeability can be rescued with additional l-Carnitine treatment. (**A**) The protective effect of l-Carnitine on lysosomal membrane stability was evaluated using acridine orange (AO) staining for lysosomal membrane permeability (LMP). The effect of PFOS on LMP was assessed using AO staining in renal tubular cells (RTCs) with or without l-Carnitine pretreatment. Cells grown on cover slips were pretreated with l-Carnitine for 24 h and then subjected to another 24-h PFOS treatment. The resulting cells were stained with AO dye for 30 min. The representative images were obtained using a fluorescence microscope and quantitated in a bar graph (100 × magnification; scale bar, 50 µm. (**B**) Transfection of a tandem GFP-RFP-LC3 reporter plasmid in RTCs was used in the evaluation of autophagy flux in PFOS-treated RTCs for 24 h with or without l-Carnitine treatment. The photos of cells with indicated treatments were taken using a fluorescent microscope (100 × magnification, scale bar, 50 µm).

We previously reported that 24-h prolonged PFOS treatment can block normal autophagic flux by increasing the accumulation of p62 and LC3BII^3^. Lysosome integrity was examined using an AO assay by assessing LMP (Fig. 6A), and the results indicated that the level of green staining was increased in RTCs treated with PFOS, indicating increased PFOS-induced LMP. The protective effect of l-Carnitine on PFOS-impaired lysosomal function was observed through AO staining for lysosome membrane stability (Fig. 6); an increased level of red AO staining was observed with the addition of l-Carnitine, indicating decreased LMP in the RTCs treated with PFOS. Furthermore, we employed a tandem GFP-RFP-LC3 autophagy flux reporter to evaluate the autophagy flux in PFOS-treated RTCs with and without l-Carnitine treatment (Fig. 6B); l-Carnitine reduced the number of LC3 green puncta but increased the number of GFP-negative/RFP-positive puncta, which are displayed in red because of the elimination of the blockade of autophagic flux in PFOS-treated RTCs.

## Discussion

In recent years, the use of perfluorinated compounds, especially PFOS, has increased considerably^26^. The resistance of PFOS to environmental and metabolic degradation has become a major concern^27,28^. PFOS induces apoptosis in the lung adenocarcinoma A549 cell line through a ROS-mediated mitochondrial dysfunctional mechanism^29^ and drainage of mitochondrial membrane potential^30^. However, the mechanisms underlying the PFOS-induced dissipation of mitochondrial membrane potential and whether these mechanisms can be protected by l-Carnitine require further verification.

Studies have reported the toxic effect of PFOS on RTCs. For example, PFOS induces oxidative stress injury due to the suppression of antioxidative enzymes (including Gpx-1, SOD1, and catalase) both in vitro and in vivo^2^. In addition, PFOS induces autophagy-associated apoptosis through ROS-mediated ERK1/2 activation, which is followed by the blockade of the normal autophagic flux in RTCs^3^. In the present study, we extended our previous research by analyzing the protective effect of l-Carnitine on autophagy-associated apoptosis and its underlying molecular mechanisms. l-Carnitine exhibits antioxidative properties by reducing the ROS level in cytosol and mitochondria. The induction of p47Phox expression can contribute to the increased cytosolic ROS level in PFOS-treated RTCs. This phenomenon can be reversed by l-Carnitine through attenuating the p47Phox level, which leads to the detection of a decreased cytosolic ROS level through the DCFDA reaction under a fluorescent microscope (Fig. 2A). In addition, the PFOS-induced increase in mitochondrial ROS reduces mitochondrial biogenesis, and l-Carnitine pretreatment can prevent this effect. Figure 3 presents the results of our Mitostress test assay; consistent with the findings of a similar study, our results revealed that PFOS induced a concentration-dependent reduction in mitochondrial bioenergenesis, demonstrating that the administration of PFOA and PFOS (50–200 μM) induced the production of ROS, dissipation of mitochondria membrane potential, and apoptosis of HepG2 cells^31^. Additional l-Carnitine treatment can reverse PFOS-induced mitochondrial function impairment by increasing ATP production, maximal respiration, and spared respiratory capacity in RTCs. In the present study, we provided potential evidence of mitophagy in PFOS-treated RTCs, including the increased ROS production in mitochondria (Fig. 2), decreased mitochondrial function (Fig. 3), and increased expression level of Bax (Fig. 4A). Additionally, PFOS reduced the levels of voltage-dependent anion channels (VDAC; Fig. 5D). These results suggest that PFOS can induce the autophagy-associated apoptosis involved in mitophagy activation. VDAC was demonstrated to be able to dictate cell death and survival, that is, a decrease in VDAC reduces cell viability. VDAC is not only related to ATP synthesis but is also involved in Bax binding to mitochondria for mitochondrial-mediated apoptosis^32,33^. We herein demonstrated that PFOS reduces VDAC and increases BAX expression in PFOS-treated RTCs, although the detailed mechanism of mitophagy remains to be determined.

ROS play crucial roles in various cellular activities, including autophagy, apoptosis, and ER stress^34,35^. We previously demonstrated that PFOS-induced ROS can result in the autophagy-associated apoptosis in RTCs, which can be suppressed by NAC (a ROS scavenger) through the suppression of PFOS-induced ERK1/2 phosphorylation. This suggests that the ERK pathway is a downstream effector of oxidative stress^3^. Herein, we extended our previous finding by demonstrating that PFOS induces autophagy-associated apoptosis in an ER-dependent manner. This finding is supported by that of another study^36^, which reported the association of ER stress with autophagy and cardiomyocyte remodeling in experimental and human atrial fibrillation. In the present study, PFOS time dependently increased IRE1α (an ER stress marker) expression, indicating ER stress induction, which could be eliminated by l-Carnitine through its antioxidative activity. Additionally, an ER stress inhibitor, 4-PBA, attenuated autophagy and subsequently apoptosis, but it did not increase the pERK1/2 level in PFOS-treated RTCs; by contrast, U0126, an inhibitor of MAPK kinases, suppressed PFOS-mediated IRE1α overexpression, suggesting that ER stress is a downstream target of the ERK pathway (Fig. 4). Our previous research demonstrated that U0126 can inhibit the ERK pathway by reversing the induced levels of pERK1/2 in PFOS-treated RTCs^3^; a growing body of evidence has indicated that U0126 exerts its antioxidative activity through the MAPK/ERK-independent pathway^37^. Therefore, a cautious interpretation is required with respect to whether U0126 executes its function through ERK inactivation or anti-oxidative activity.

Regarding the signaling pathway that is involved in autophagy induction, the present study demonstrated that PFOS induced ERK1/2 and ER stress activation, leading to autophagy induction with an increased expression of Beclin and LC3BII; this finding is consistent with that of another study, that is, the activation of the MAPK pathway (e.g., ERK1/2 activation) is involved in ER stress, cell proliferation, cell cycle arrest, and autophagy^38^. In addition to the PFOS-induced pERK1/2 that is involved in autophagy activation, we revealed the inhibition of pmTOR and pAKT in PFOS-mediated RTC autophagy, which is an effect that can be reversed through l-Carnitine pretreatment (Fig. 5A). Furthermore, our results revealed that l-Carnitine reduced PFOS-mediated autophagy through the suppression of ROS levels, which is supported by the results of another study that revealed that l-Carnitine attenuates autophagy induction by suppressing ROS production in C2C12 cells^39^. However, l-Carnitine can also induce autophagy in C2C12 cells with high-fat-diet-mediated glucose intolerance and insulin resistance^40^. Therefore, the effect of l-Carnitine on autophagy induction in a given type of cell can differ depending on the circumstances, it can protect cells from damage either by increasing or suppressing autophagy induction in C2C12 cells. These results further contribute to the complexity of the findings regarding the dual nature of autophagy in cells and organisms. Similarly, cisplatin, a chemotherapy chemical, caused toxic complications of autophagy and cell death in mouse renal tubular epithelial EpiCM-a cells through ERK1/2 activation, whereas aristolochic acid I attenuated cell apoptosis through pERK1/2-mediated autophagy activation in rat RTCs (NRK-52E)^41^. Thus, for a given type of cell, autophagy can either cause cell death or maintain cell viability through a similar signaling pathway. A clinical study reported that the autophagy-mediated apoptosis caused by ROS production or ERK1/2 activation allows resveratrol to exert its antitumor effect on the apoptosis of human colon cancer cells through ROS-triggered autophagy^42^. Similarly, triptolide was implicated in the treatment of the breast cancer cell line MCF-7 through the induction of autophagy-mediated cell death by increasing ERK1/2 phosphorylation^43^.

In the present study, a concentration of 100 µM PFOS is higher than the average level of exposure; given the short duration of PFOS exposure (e.g., 1, 4, 6 and 24 h) to RTCs, the concentration that was used in our study was approximately four times that of the PFOS concentration that fluorochemical production workers are exposed to. Other studies employed higher concentrations of PFOS (up to 300 µM) to study apoptosis and autophagy activation in hepatocytes^31,44,45^. Notably, l-Carnitine exhibited a protective effect on PFOS-treated RTCs, even though the concentration of PFOS being examined in this experimental setting was fourfold higher than the typical exposure rate; this finding indicates the potential of l-Carnitine in clinical applications.

## Conclusions

**Figure 7:**
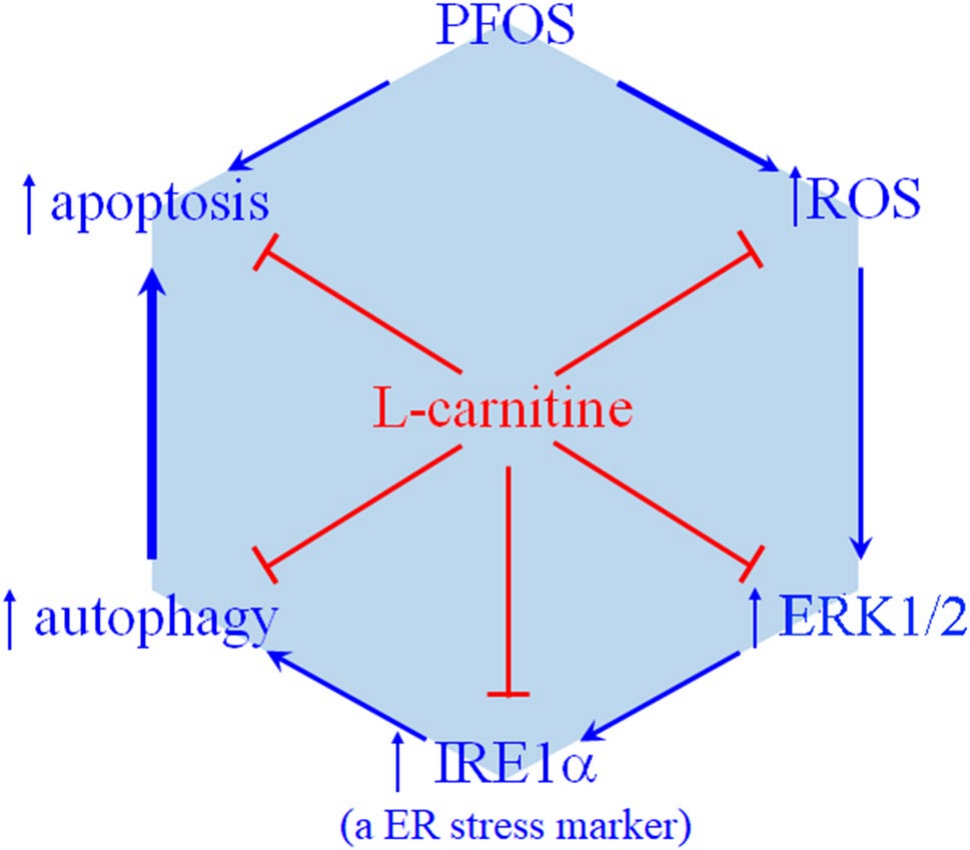
Summarized scheme of the mechanism underlying the protective effect of l-Carnitine on autophagy-associated apoptosis in perfluorooctanesulfonate (PFOS)-treated renal tubular cells (RTCs), which produced an antiautophagic effect through its antioxidative activity. The protective effect of l-Carnitine on PFOS-mediated apoptosis involves the sequential reduction of ERK1/2 activation, ER stress, autophagy activation, and finally apoptosis. The sequential relationship of ROS to induce ERK1/2 phosphorylation to increase the inositol-requiring enzyme 1α level and then ER stress to activate autophagy was verified using their respective inhibitors, namely U0126 (a MAPK kinases inhibitor) and 4-PBA (an ER stress inhibitor).

ROSs play an essential role in the autophagy-associated apoptosis in PFOS-treated RTCs, which is associated with the impairment of mitochondrial bioenergetics and the effects of uncoupling on reduced ATP synthesis. Notably, l-Carnitine can reverse these effects by maintaining a normal mitochondrial function and restoring cellular viability. The findings of this study are presented schematically in Fig. 7. The novel finding of the present study is that l-Carnitine protects RTCs from PFOS-mediated apoptosis by inhibiting the induction of ROS, pERK1/2, and IRE1α and then causing autophagy activation. Collectively, l-Carnitine treatment reverses PFOS-mediated oxidative stress in cytosol and mitochondria through its antioxidative activity and subsequently protects RTCs from autophagy-associated apoptosis.

## Supplementary Information

Supplementary Information.
