# Supplementary material for: l-Carnitine reduces reactive oxygen species/endoplasmic reticulum stress and maintains mitochondrial function during autophagy-mediated cell apoptosis in perfluorooctanesulfonate-treated renal tubular cells
Source: Sci Rep. 2022 Mar 18;12:4673. doi: 10.1038/s41598-022-08771-3 (PMC8933466; doi:10.1038/s41598-022-08771-3)
Supplement: Supplementary file 1 — Supplementary Information. [file 41598_2022_8771_MOESM1_ESM.pdf]

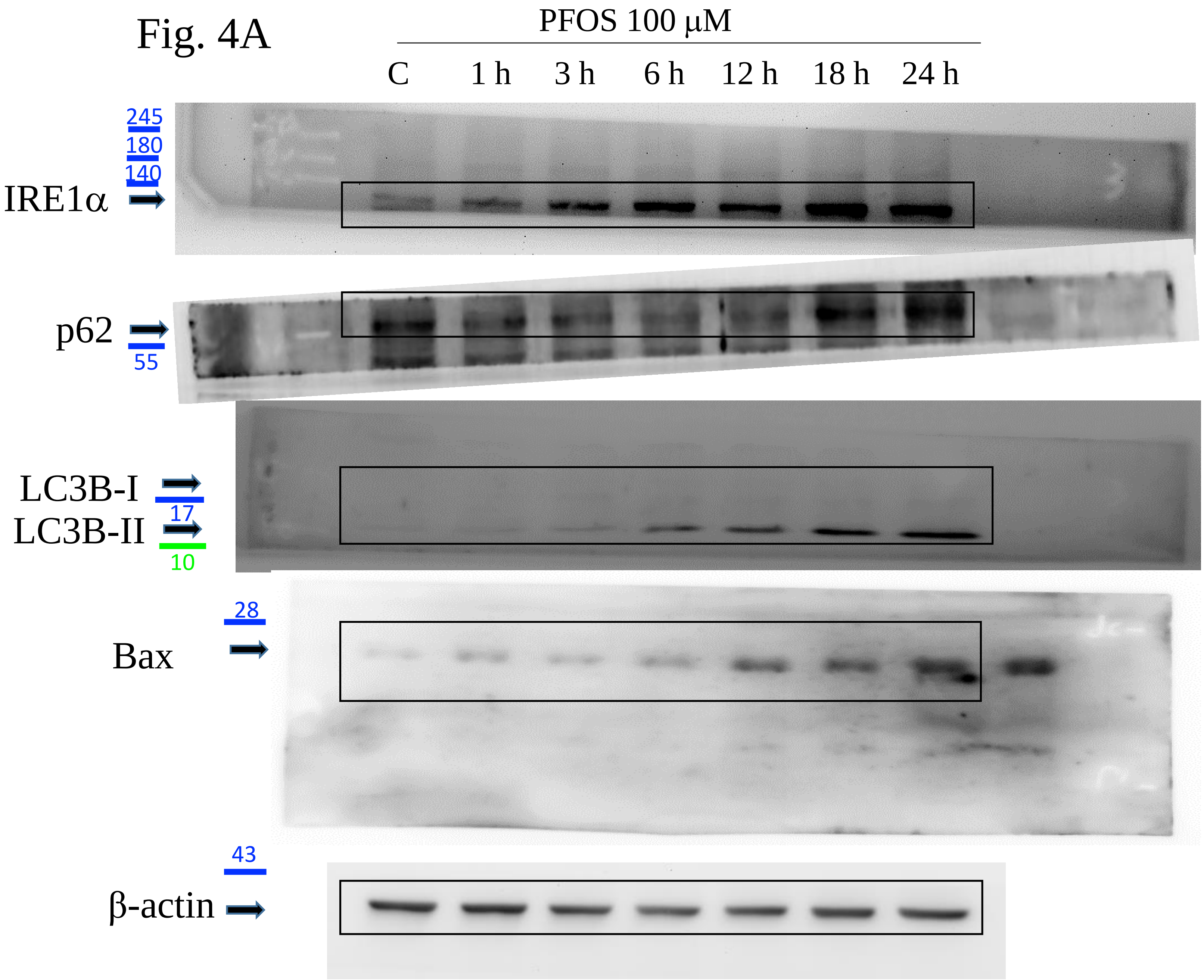
Fig. 4A
PFOS 100 μM
C
1 h
3 h
6 h
12 h
18 h
24 h
245
180
140
IRE1α
p62
55
LC3B-I
17
LC3B-II
10
28
Bax
43
β-actin

Fig 4C_2

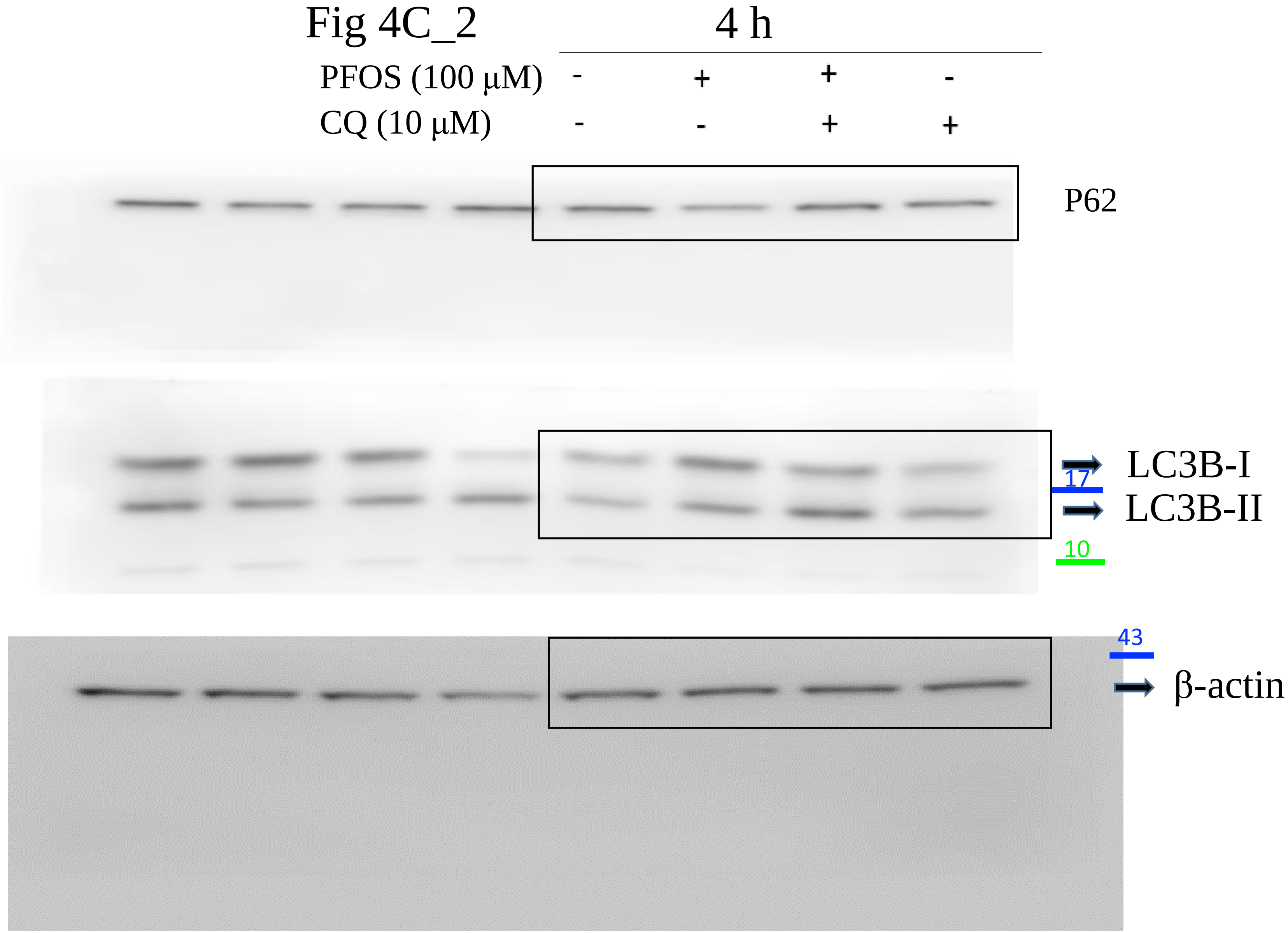

Fig 4D_3

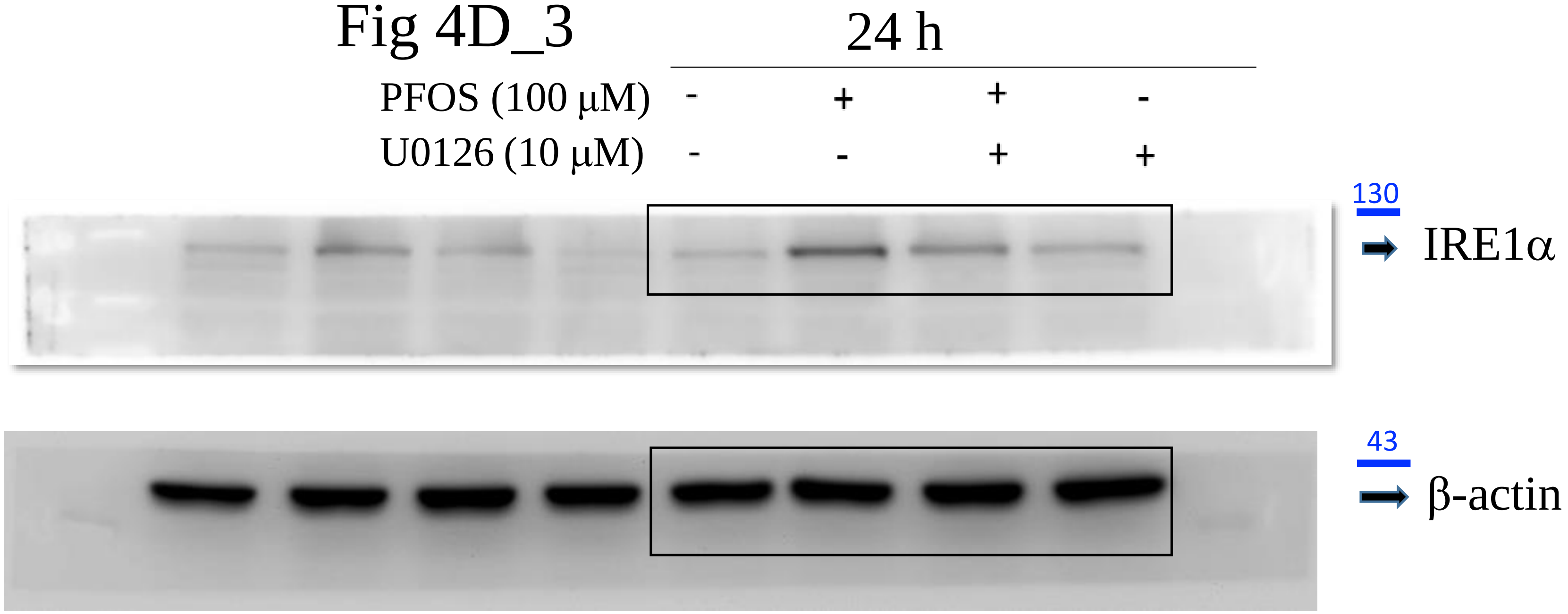

Fig 4E_4

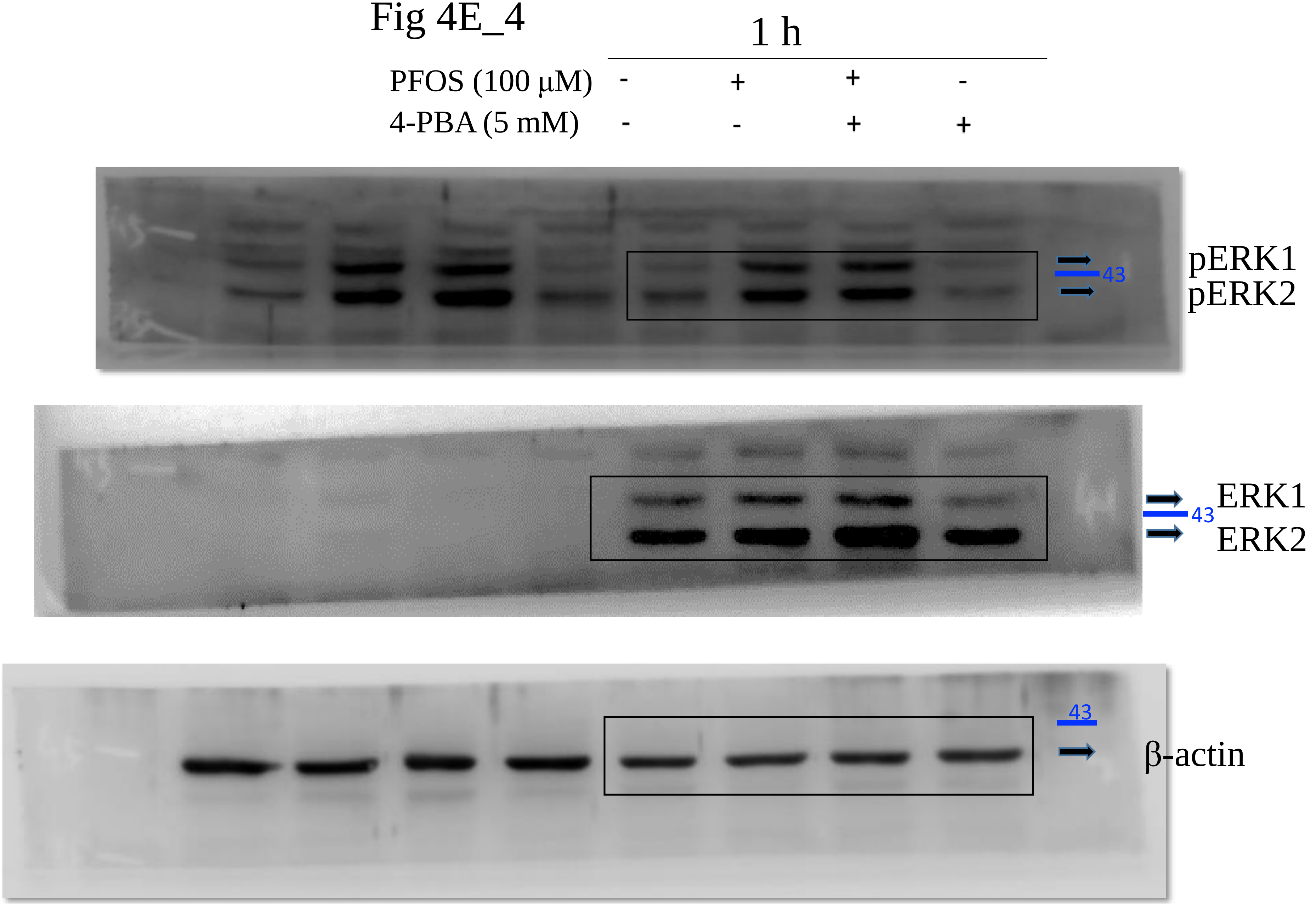

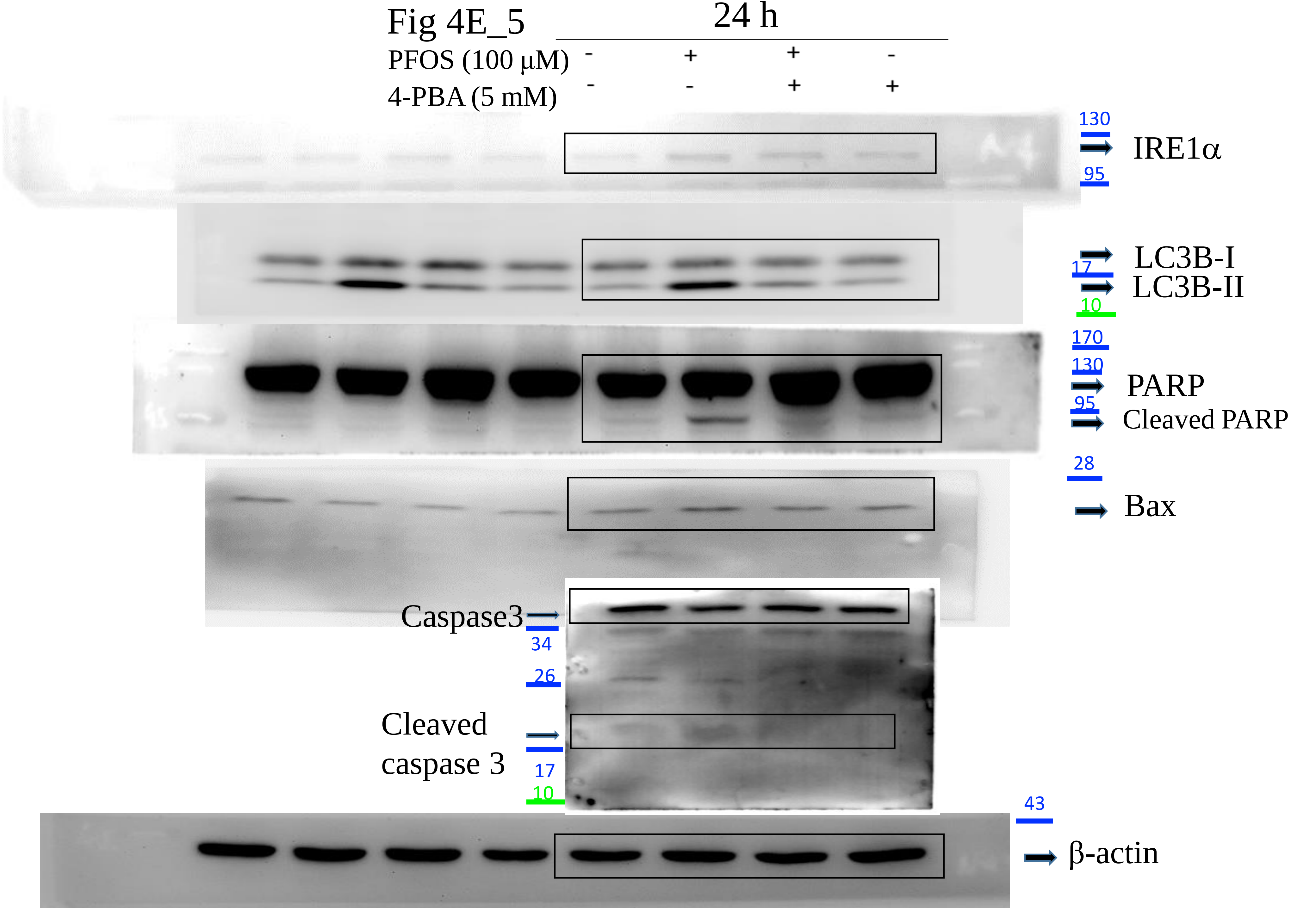

Fig 4E_5
24 h
PFOS (100 μM) - + + -
4-PBA (5 mM) - - + +
130
95
IRE1α
LC3B-I
17
LC3B-II
10
170
130
PARP
95
Cleaved PARP
28
Bax
Caspase3
34
26
Cleaved caspase 3
17
10
43
β-actin

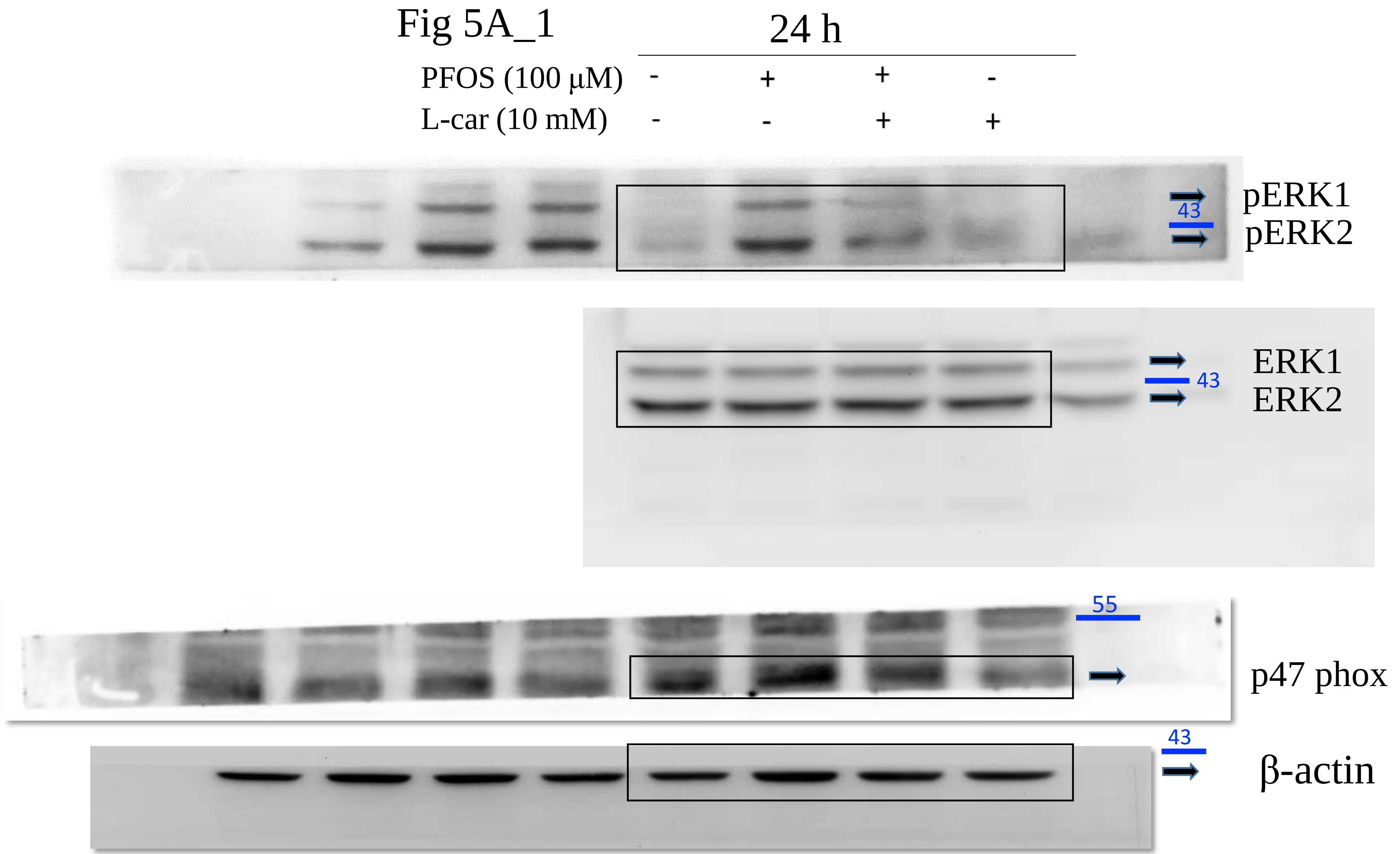
Fig 5A_1
24 h
PFOS (100 μM) - + + -
L-car (10 mM) - - + +
pERK1
pERK2
43
ERK1
ERK2
43
p47 phox
55
β-actin
43

Fig 5A_2

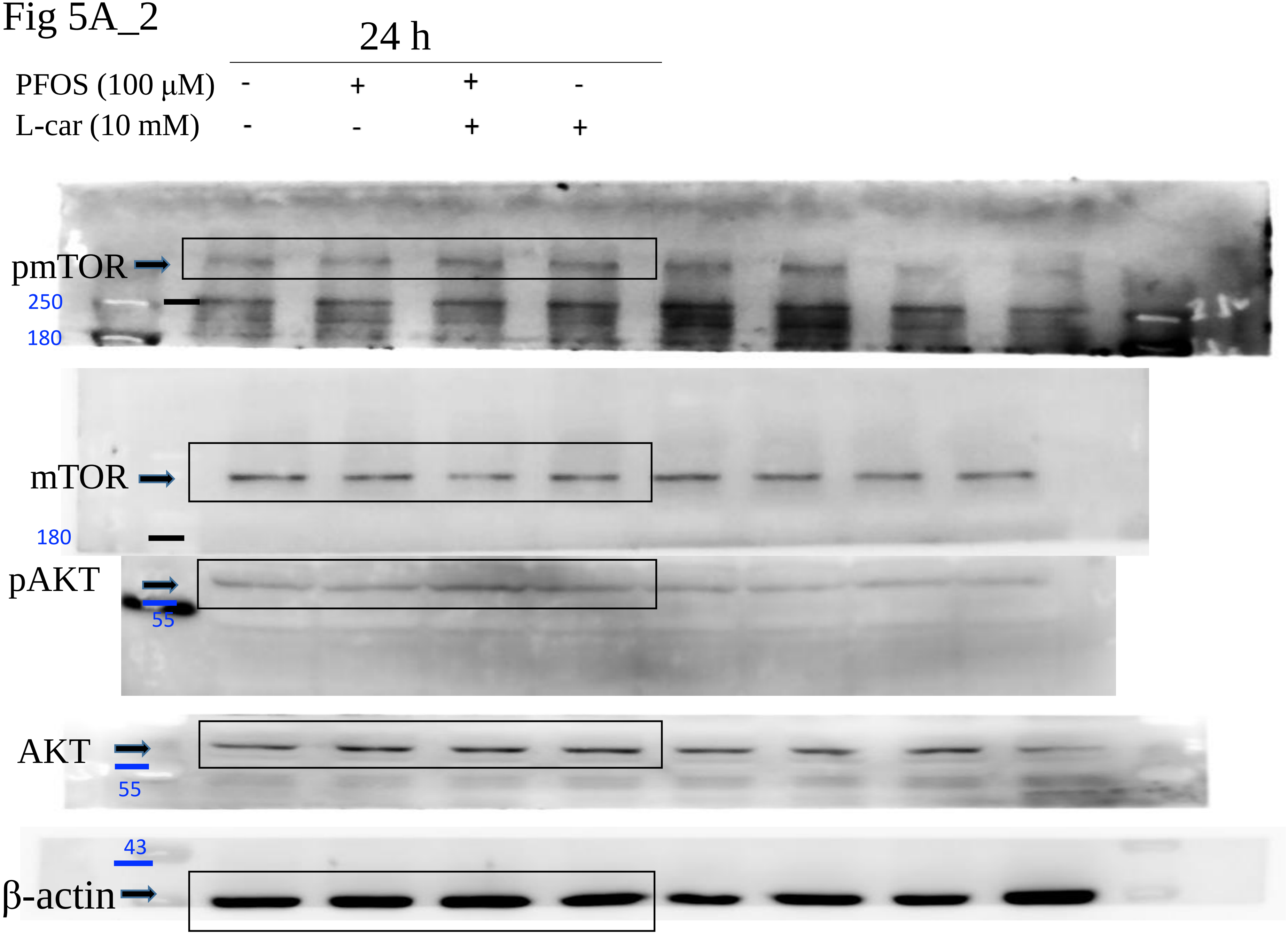
24 h
PFOS (100 μM) - + + -
L-car (10 mM) - - + +
pmTOR
250
180
mTOR
180
pAKT
55
AKT
55
43
β-actin

Fig 5B_3

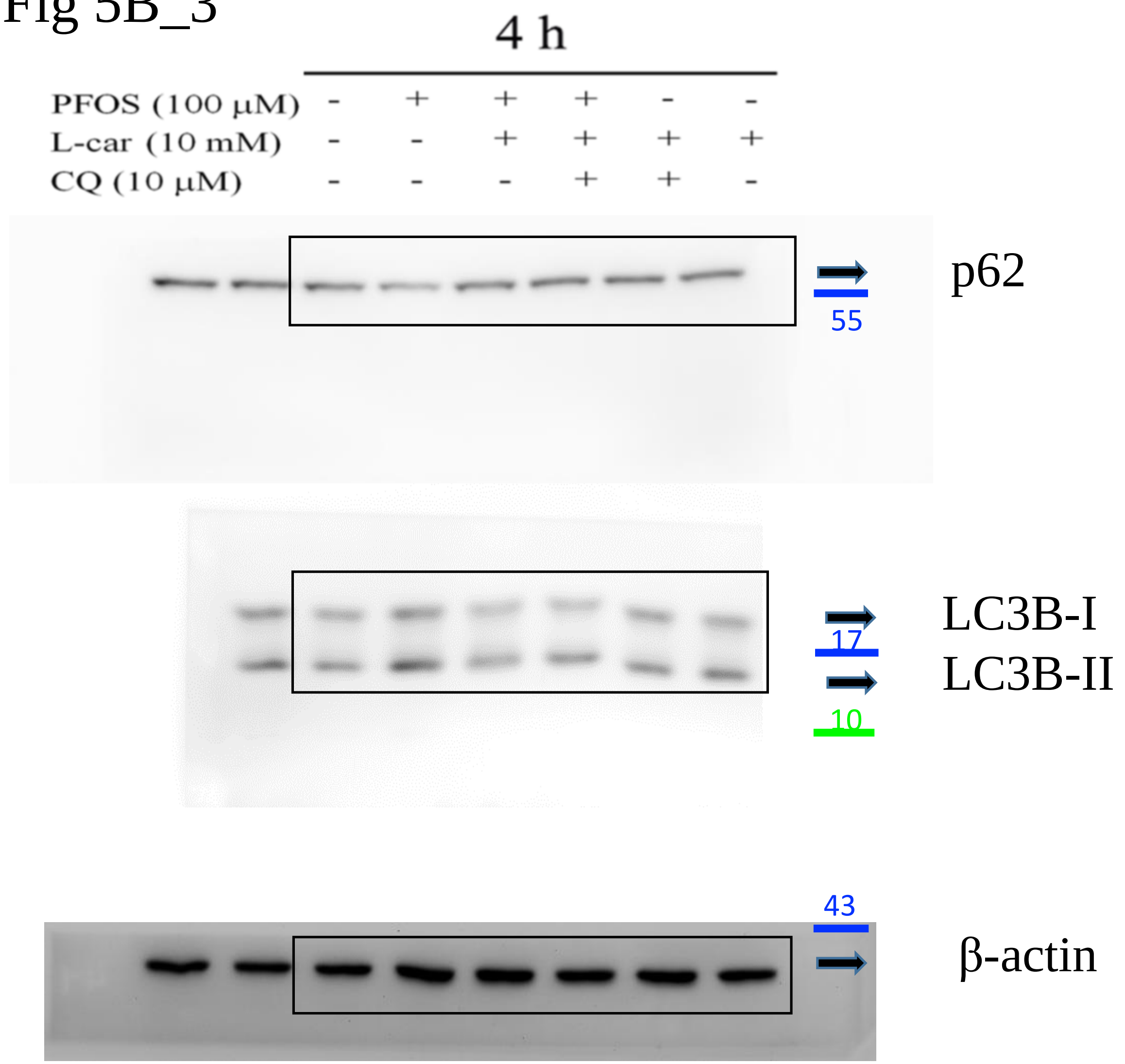
4 h
PFOS (100 μM) - + + + - -
L-car (10 mM) - - + + + +
CQ (10 μM) - - - + + -
p62
55
LC3B-I
17
LC3B-II
10
43
β-actin

Fig 5C_4

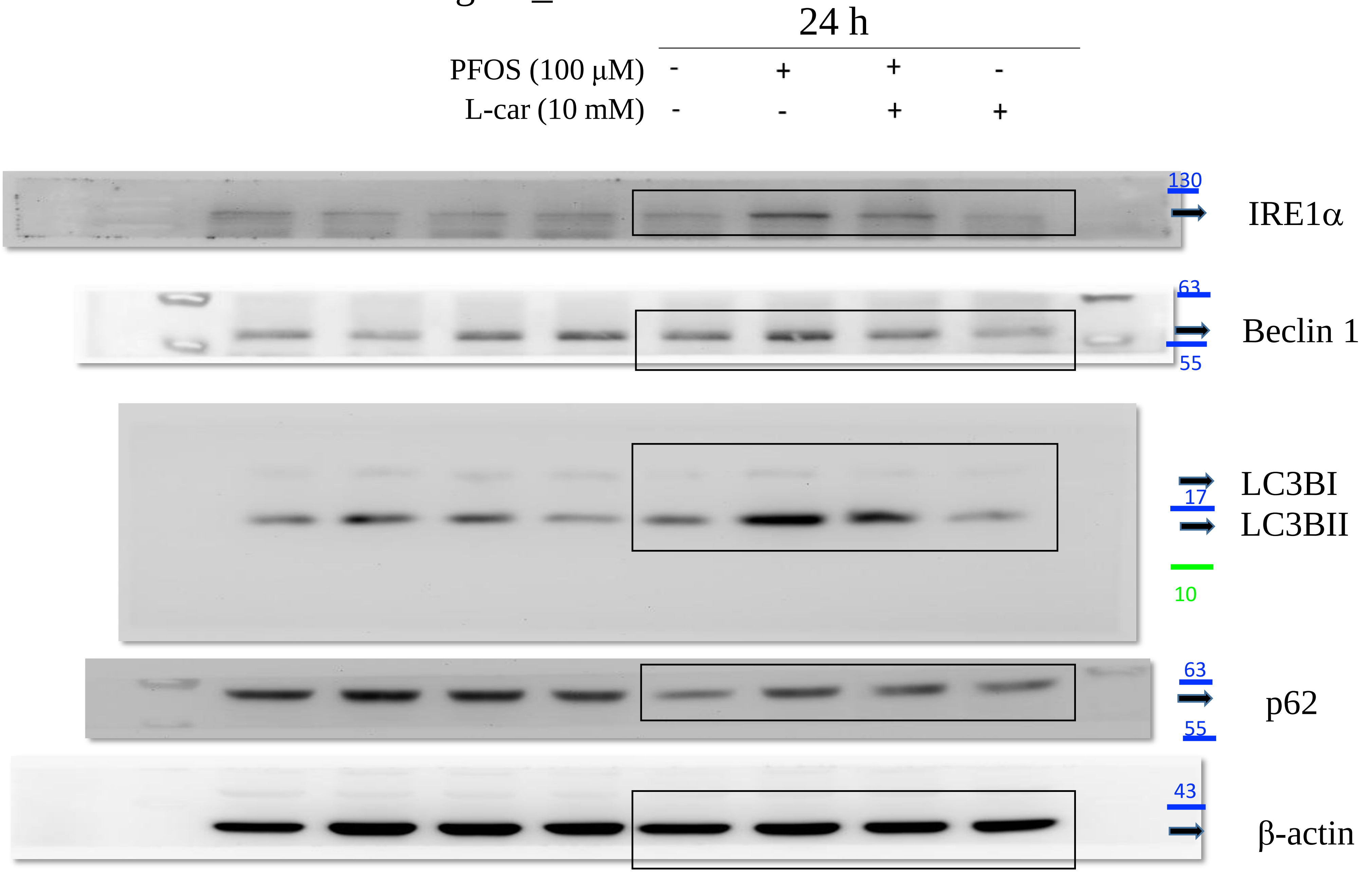
24 h
PFOS (100 μM) - + + -
L-car (10 mM) - - + +
130
IRE1α
63
Beclin 1
55
LC3BI
17
LC3BII
10
63
p62
55
43
β-actin

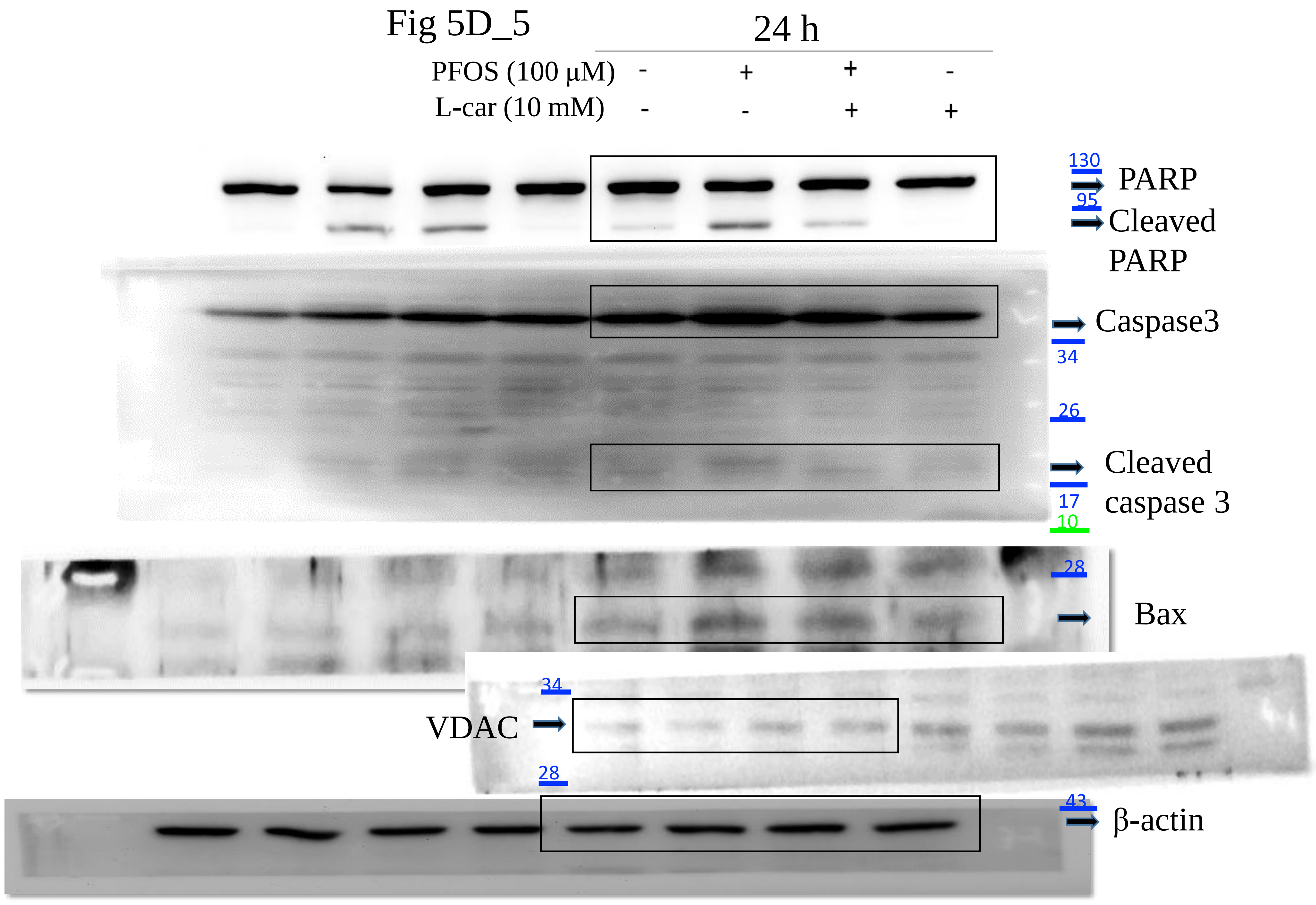
Fig 5D_5
24 h
PFOS (100 μM) - + + -
L-car (10 mM) - - + +
130
PARP
95
Cleaved PARP
Caspase3
34
26
Cleaved caspase 3
17
10
28
Bax
34
VDAC
28
43
β-actin
